# Test‐Retest Reliability of Physiological Resilience During and After Prolonged Moderate‐Intensity Running in Well‐Trained Runners

**DOI:** 10.1002/ejsc.70178

**Published:** 2026-04-28

**Authors:** Timi Malinen, Olli‐Pekka Nuuttila, Pekka Matomäki, Arja Uusitalo, Heikki Kyröläinen

**Affiliations:** ^1^ Faculty of Sports and Health Sciences University of Jyväskylä Jyväskylä Finland; ^2^ UKK Institute for Health Promotion Research Tampere Finland; ^3^ Paavo Nurmi Centre and Unit for Health and Physical Activity University of Turku Turku Finland; ^4^ Department of Sports and Exercise Medicine Clinicum University of Helsinki Helsinki Finland; ^5^ Helsinki Clinic for Sports and Exercise Medicine Foundation for Sports and Exercise Medicine Helsinki Finland

**Keywords:** durability, endurance performance, endurance running, fatigue resistance, reliability

## Abstract

In previous research, physiological resilience has been measured as deterioration of the physiological profile during prolonged exercise. This study aimed to evaluate the test–retest reliability of physiological resilience during prolonged moderate‐intensity running. Physiological profile of 26 well‐trained endurance runners (10 females) was tested in nonfatigued state as well as during and after two identical ∼2.5‐h long physiological resilience tests at ∼89% of VT1 (ventilatory threshold 1) speed within an average period of 13 days. Reliability was assessed with intraclass correlation coefficients (ICC), coefficient of variations (CV%), and typical errors (TE and TE%). Change in maximal speed achieved in the incremental test (sPeak) was the most reliable metric and showed good reliability (ICC: 0.81 and TE: 1.8). The drifts in running economy (RE) and the heart rate (HR) had moderate to good reliability in the second half of the trial (ICC: 0.52–0.80 and TE 1.7–2.4). The changes in maximal oxygen uptake (VO_2max_) and ventilatory thresholds (VTs) had poor reliability (ICC: 0.07–0.36 and TE: 3.5–4.6). However, the absolute values of these variables demonstrated good to excellent reliability in fatigued state (ICC > 0.83, TE% < 5.2%, and CV% < 4.1%) even though they significantly deteriorated. Determining changes in sPeak and drifts in RE and HR appears to be the most reliable method to measure physiological resilience. In contrast, measurement of the physiological profile remains reliable in fatigued state for every variable. Longer or more demanding protocols may be required to obtain greater reliability for deterioration of the physiological profile.

## Highlights

- The physiological profile can be measured with same reliability during and after prolonged moderate‐intensity running as in a nonfatigued state
- Measurement of physiological resilience (ability to resist deterioration of the physiological profile) is only reliable when measuring the change in sPeak or drifts in the heart rate and running economy but not when measuring VO_2max_ or ventilatory thresholds
- It appears that the testing protocol needs to be demanding enough such that the deterioration exceeds the variability of the measurement to ensure reliability.

## 1 Introduction

Endurance running performance is typically determined by maximal oxygen uptake (VO_2max_), lactate or ventilatory thresholds (LTs or VTs), and running economy (RE) (Joyner 1991; Joyner and Coyle 2008). Traditionally, the physiological profile, defined by these measures, has been assessed in a nonfatigued state. However, this profile has been shown to deteriorate during prolonged exercise, and the extent of deterioration is highly individual (Jones and Kirby 2025). It has been proposed that the measurement of the physiological profile in a fatigued state would help better predict performance (Noordhof et al. 2021). The ability to resist the deterioration during prolonged exercise has been called “physiological resilience” or “durability” in recent years (Jones 2024; Maunder et al. 2021) and has been proposed as the fourth independent determinant of endurance performance and an important factor during endurance competitions (Jones 2024). The terminology has been under scientific debate (Meixner et al. 2025). It has been proposed that durability means the ability to resist deterioration of performance speed/power, while physiological resilience as the ability to sustain physiological functions (e.g., VO_2max_ and RE) (Zanini et al. 2025a). For clarity, the current authors have decided to use term “physiological resilience” as an umbrella term in the current paper.

As the concept of physiological resilience is new, no standardized protocol exists to measure it. A recent review by Hunter et al. (Hunter, Maunder, et al. 2025) concluded that physiological resilience has been assessed using various physiological and performance approaches. These include changes in LTs (Nuuttila et al. 2024), VTs (Barrett and Maunder 2025; Gallo et al. 2024), RE (Zanini et al. 2024, 2025a), physiological drifts (e.g., heart rate [HR] or oxygen uptake [VO_2_]) (Matomäki et al. 2023), VO_2max_ (Unhjem 2024), or multiple variables (Zanini et al. 2025a, 2025b; Hunter and Muniz‐Pumares 2025; Evans et al. 2025) during prolonged moderate‐ or heavy‐intensity running or cycling. Changes in the heart rate variability variable and detrended fluctuation analysis alpha‐1 (DFA‐ α1) have also been studied as a marker of resilience (Nuuttila et al. 2024). Although not directly linked to physiological resilience, neuromuscular fatigue induced by prolonged endurance exercise has been shown to result in decreased maximal voluntary contraction (MVC) and voluntary activation (Millet et al. 2018) as well as reduced reflex sensitivity (Avela et al. 1999) affecting performance negatively. Although the reliability of the physiological profile and neuromuscular performance is well established in nonfatigued states (Blagrove et al. 2017; Gaskill et al. 2001; Lourenço et al. 2011; Shaw et al. 2013), it remains unclear how reliability is affected when measured during or after prolonged exercise. Understanding whether the measurement of these variables affecting endurance performance remain reliable during prolonged running is crucial for future mechanistic studies on physiological resilience. Because the ability to sustain the physiological profile is multifactorial and influenced by various biological systems (Jones 2024), determining whether these variables can be measured reliably is an important question when monitoring athlete performance or developing related research.

To the best of our knowledge, only one study has examined the reliability of fatigued state physiological profile or physiological resilience assessments during prolonged running (Zanini et al. 2025b). Zanini et al. (2025b) reported that RE (as oxygen cost or energy cost [EC]), respiratory exchange ratio (RER), ventilation, and HR showed high reliability, while blood lactate levels (BLa) and the rate of perceived exertion showed moderate reliability during 90 min of heavy‐intensity running. In cycling, (Clark et al. 2018) found that critical power measured after 2 h of heavy‐intensity cycling is highly reliable for assessing physiological resilience, and similar results have been observed for maximal mean power output during field‐based assessments (Mateo‐March et al. 2025). According to our knowledge, no studies have assessed the reliability of postexercise VO_2max_, VT2, and VT1 or the reliability of the deterioration of the physiological profile during prolonged running. Although measurement of the physiological profile in fatigued state is important as it separates different levels of athletes (Leo et al. 2021), it does not account for the deterioration in the profile.

In most of the previous studies, physiological resilience has been measured as the percent change in a chosen variable (Nuuttila et al. 2024; Barrett and Maunder 2025; Gallo et al. 2024; Zanini et al., 2025a, 2025b) highlighting the importance of understanding the reliability of this method (Faude et al. 2025). To understand the reliability of the change in the physiological profile, there is a need to know whether the reported change (signal) exceeds the error (noise) of the measurement. Reliable protocols for assessing deterioration in the physiological profile would allow researchers to measure improvement in physiological resilience in interventional studies and support broader performance monitoring for athletes and coaches.

The purpose of this study was to examine the test–retest reliability of fatigued physiological profile and its deterioration (physiological resilience) in well‐trained endurance runners. Reliability was assessed in VO_2max_, VTs, RE, HR drift, and neuromuscular performance during and after prolonged moderate‐intensity running. We hypothesize that changes in the physiological profile will be less reliable than the absolute fatigued profile due to compounded measurement error.

## 2 Materials and Methods

### 2.1 Participants

Twenty six well‐trained endurance runners (16 males and 10 females) volunteered the study and gave written informed consent. Participants were categorized as Tier 2 or 3 (McKay et al. 2022). Eligibility criteria were (i) age: 18–45, (ii) running ≥ 3 times per week during the previous 3 months, (iii) half‐marathon time under 120 min or an equivalent performance in a longer distance running race, a triathlon, or an orienteering event in the last 12 months, and (iv) free from musculoskeletal injuries, metabolic and cardiovascular diseases, and upper respiratory tract infections within the last 2 weeks. The study was approved by the ethics committee of University of Jyväskylä (1581/13.00.04.00/2024) and conducted in accordance with the Declaration of Helsinki. Prior to the first laboratory visit, participants underwent through a resting electrocardiogram, verified by a licensed physician, and completed training history and health questionnaires to confirm eligibility. Characteristics of the participants are presented in Table 1.

**TABLE 1:** Mean (± SD) characteristics of the participants.

Measure	All (*n* = 26)	Males (*n* = 16)	Females (*n* = 10)
Age (years)	30.7 ± 6.6	31.8 ± 7.3	29.1 ± 4.7
Height (cm)	175.5 ± 10.0	181.9 ± 6.5	165.1 ± 4.5
Body mass (kg)	72.4 ± 11.6	79.8 ± 7.2	60.5 ± 6.2
VO_2max_ (ml·kg^−1^ min^−1^)	55.4 ± 7.3	57.2 ± 7.5	52.4 ± 6.0
sPeak (km/h)	17.9 ± 1.7	18.6 ± 1.6	16.8 ± 1.2
sVT2 (km/h)	15.2 ± 1.8	15.8 ± 1.8	14.3 ± 1.5
sVT1 (km/h)	12.0 ± 1.8	12.3 ± 1.9	11.6 ± 1.4

Abbreviations: sPeak, maximal speed achieved in VO_2max_ test; sVT2, speed at the second ventilation threshold; sVT1, speed at the first ventilation threshold; VO_2max_, maximal oxygen uptake.

### 2.2 Study Design

The participants visited the laboratory on three occasions. Visit 1 included an incremental running test and VO_2max_ test to assess the physiological profile in a nonfatigued state. Visits 2 and 3 consisted of identical physiological resilience tests. The average interval between Visits 1 and 2 was 5.7 ± 2.5 days and between Visits 2 and 3 was 7.1 ± 1.6 days. Visits 2 and 3 were performed at the same time of day (± 2 h). All measurements were done in the same laboratory using the same treadmill (Telineyhtymä Oy, Kotka, Finland) and in the same environmental conditions (temperature: 21.2 ± 0.8°C and relative humidity: 26.8 ± 7%) Participants were asked to refrain from caffeine for 12 h and from alcohol and intense exercise for 24 h before each visit. Participants were instructed to maintain the same diet and exercise routine for the 24 h preceding both physiological resilience tests and wear similar clothing and same footwear in all trials.

#### 2.2.1 Visit 1: Incremental Treadmill Test and VO_2max_ Test

During Visit 1, participants completed an incremental test to assess VT1 and a separate VO_2max_ test to assess VO_2max_ and VT2 in a nonfatigued state. Upon arrival at the laboratory, the height was measured to the nearest 0.1 cm and weight in running clothes without shoes to the nearest 0.1 kg. Participants then completed a 3‐min warm‐up at the starting speed of the incremental test. The incremental test consisted of 3‐min stages where speed increased by 1 km·h^−1^ in every stage (Weltman et al. 1990), while treadmill inclination was kept at 1% throughout the test (Jones and Doust 1996). The starting speed was 7–10 km·h^−1^, based on the participants self‐reported race results. After each stage, the treadmill was stopped to collect a fingertip blood sample. The test continued until the RER reached and stayed continuously over 1.00 during the final minute of the stage to ensure the achievement of VT1 (Pallarés et al. 2016). After the first test, participants rested for 10 min prior to completing a VO_2max_ test. The starting speed for the VO_2max_ test was set 2 km·h^−1^ higher than in the incremental test and was increased by 1 km·h^−1^ every minute until volitional exhaustion occurred. During both tests, HR (Polar H10, Polar Oy, Kempele, Finland) and respiratory gases (Jaeger VyntusTM CPX, CareFusion Germany 234 GmbH, Hoechberg, Germany) were continuously monitored. An electric fan was placed approximately 2 m in front of the participants, directed at the torso and the lower body with constant speed throughout.

#### 2.2.2 Visit 2 and 3: Physiological Resilience Test

During the second and third visits, participants completed identical physiological resilience tests. Upon arrival at the laboratory, weights were measured in running clothes with and without shoes. Participants then performed the same warm‐up as in the first visit, followed by countermovement jumps (CMJs) on a contact mat. Two warm‐up jumps and three maximal jumps were performed with 15‐s rest intervals. Participants were instructed to descend to a self‐selected depth, jump maximally upward with hands on hips, and land with straight legs on the midfoot. After CMJs, participants performed an isometric leg press test at a knee angle of 108° using a custom‐made isometric leg press. Following warm‐ups with 50%, 75%, and 90% of their self‐estimated maximal force, they executed three maximal contractions with 30 s of rest between trials.

**FIGURE 1:**
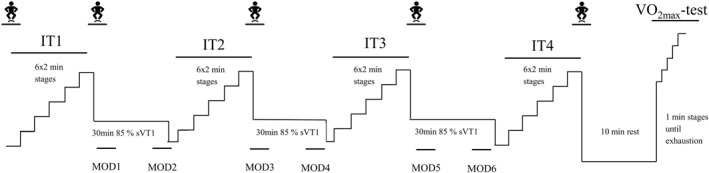
The physiological resilience test protocol. IT, incremental test consisting of 6 2‐min stages; Jumper, countermovement jumps and weight measurement; MOD1–6, timepoint of moderate intensity running; sVT1, speed at ventilatory threshold 1; VO_2max_, maximal oxygen uptake.

The physiological resilience test protocol (Figure 1) was adapted from previous studies involving 90–120 min of moderate‐intensity exercise (Nuuttila et al. 2024; Barrett and Maunder 2025; Stevenson et al. 2022) and protocols using incremental tests to measure time‐dependent changes in VT1 (Gallo et al. 2024). The test consisted of four short incremental tests (IT1‐4) to measure VT1 and three 30‐min moderate‐intensity running bouts. Each IT included six two‐minute stages beginning at a speed 2.4 km·h^−1^ below the sVT1, measured during Visit 1, with speed increasing by 0.8 km·h^−1^. The IT protocol was chosen to limit the time spent in the heavy‐intensity domain. During each of the ITs, the treadmill ran continuously. Respiratory gases were continuously monitored. After the final stage, participants stood for 60 s for BLa sampling, after which the mask was removed, and three maximal CMJs were performed. Participants were then reweighed with shoes on.

Between ITs, participants completed 30 min of moderate intensity running (MOD) at 85% of sVT1 from Visit 1. Eighty‐five percent of sVT1 was chosen to average the intensity to about 90% of VT1 with ITs. During MODs, respiratory gases were recorded during the first and last 5 min of each MOD from which RE was determined. The results in the different timepoints will be referred to as MOD1–MOD6 in the following sections (time after starting the test: 14–19 min [MOD1], 39–44 min [MOD2], 59–64 min [MOD3], 84–89 min [MOD4], 104–109 min [MOD5], and 129–134 min [MOD6] from the start). Participants removed the mask while running, but the treadmill was stopped for ∼15 s–5.5 min before the end of each MOD after which they put back the mask back. After the MOD, participants stood still for 1.25 min for BLa sampling, after which the next IT started. Participants were permitted to drink water ad libitum when not wearing the mask but were not allowed to consume exogenous energy. Participants were permitted to use the toilet during the MODs, but the stoppage was timed, and the full minutes were added to the MOD to ensure 30 min of running. Similar to Visit 1, an electric fan was placed in front of the participant.

After completing four ITs and three MODs, participants rested for 10 min after which they performed the same VO_2max_ test as during the first visit, followed by the isometric leg press test. At the midpoint of the 10‐min recovery period, participants rinsed their mouths with 200 mL (2.8 ± 0.5 mL·kg^−1^) of a 6% carbohydrate solution, whose quantity is similar as that previously used (Pottier et al. 2010). They were instructed to repeat the following procedure, take a mouthful at time, rinse for 5 s, and expectorate, until the solution was rinsed. Participants were advised not to swallow the solution. The procedure was repeated identically during both tests. The purpose of the rinse was to enhance motivation during the VO_2max_ test and minimize the influence of central fatigue, without ingesting carbohydrates, as the study aimed to measure maximal physiological capacity. Carbohydrate mouth rinsing has been shown to activate brain regions associated with motivation (Painelli et al. 2010), though it has not been shown to improve maximal performance during 15‐min maximal exercise (Jeffers et al. 2015) or incremental exercise test with 1‐min stages (Pires et al. 2024).

### 2.3 Analyses of Physiological Profile and Resilience

#### 2.3.1 Maximal Measures

VO_2max_ was defined as the highest 30‐s running average. Maximal speed from the VO_2max_ tests (sPeak) was calculated as follows: last completed stage (km·h^−1^) + (duration of unfinished stage [in seconds])/(60 s) x 1 km·h^−1^.

#### 2.3.2 Ventilatory Thresholds

VT1 was identified from the incremental tests as VO_2_ where increase in VCO_2_, Ve·VO_2_^−1^, and excess CO_2_ occurred (Gaskill et al. 2001). VT2 was determined from the VO_2max_ test as VO_2_ where an increase in Ve·VCO_2_^−1^ and more rapid increase in Ve·VO_2_^−1^ occurred (Keir et al. 2022). These VO_2_ values were converted to corresponding speeds (sVT1 and sVT2) by linear fit of speed and VO_2_. The linear fit was based on end stage average VO_2_ (60 s in the threshold test, 30 s in the ITs, and 20 s in the VO_2max_‐tests), with averaging periods determined by the different lengths of the stages. Thresholds were independently evaluated by two blinded experienced researchers. If their analyzed VO_2_ values differed by ≤ 2 mL·kg^−1^ min^−1^, the mean was used; if > 2 mL·kg^−1^ min^−1^, they reassessed together until agreement was reached. Although the VT1 protocol in Visit 1 (3‐min stages and 1‐km·h^−1^ increments) differed from the physiological resilience tests (2‐min stages and 0.8‐km·h^−1^ increments), the intraclass correlation coefficient (ICC) between sVT1 from Visit 1 and the first stage of the first physiological resilience test was 0.94 (95% CI: 0.87–0.97), with a coefficient of variation of 2.8%, indicating excellent agreement between the methods.

#### 2.3.3 Running Economy

RE was measured from the MODs and expressed as EC (kcal·kg^−1^·km^−1^) (Zanini et al. 2024), which accounts for both oxygen cost and substrate utilization (Blagrove et al. 2017). Results for OC are presented in Supporting Information S1. Utilization of proteins was assumed negligible. Fat and carbohydrate utilization (g·min^−1^) were calculated according to Jeukendrup and Wallis (2005) (Jeukendrup and Wallis 2005), and energy expenditure was calculated by multiplying fat and carbohydrate utilization by 9.75 and 4.07 kcal, respectively. The body mass used in the calculation was the value recorded immediately before each 30‐min MOD where RE was assessed. VO_2_ and VCO_2_ were averaged over the final 2 min of each MOD. The gas analyzer was calibrated before and after each test. If calibration drift exceeded ± 2%, VO_2_ and VCO_2_ values were linearly corrected.

#### 2.3.4 Heart Rate and Heart Rate Variability

HR and DFA‐α1 were measured during the last 2 min of each MOD and were analyzed using Kubios HRV Scientific software (version 4.0) with default settings (Van Hooren et al. 2025) and automatic beat correction (Lipponen and Tarvainen 2019). Four tests exceeded the noise threshold of 3% and were excluded. Thus, HR and DFA‐ α1 analysis included 22 participants. DFA‐α1 was chosen as a HRV variable as it has been proposed to be linked to the deterioration of the physiological profile during prolonged running (Nuuttila et al. 2024).

#### 2.3.5 CMJ Height and Maximal Voluntary Contraction (MVC)

CMJ height was calculated from the flight time as follows: (9.81·(flight time)^2^)·8^−1^ (Bosco et al. 1983). MVC was analyzed as the highest force produced during the isometric leg press test using Signal 4.11 software (Cambridge Electronic Design Ltd., Milton, United Kingdom).

### 2.4 Statistical Analysis

All results are reported as mean ± standard deviation. Normality was assessed using the Shapiro–Wilk test, histograms, and Q–Q plots. Changes (Δ%) in VO_2max_, sPeak, sVTs, RE, CMJ, and HR were calculated as follows: ([POST‐PRE]/PRE) × 100%. Changes in VT1 were calculated in two ways: by using the IT1 as the baseline and by using the VT1 from visit 1 as the baseline. Change in DFA‐α1 was calculated as absolute change (POST‐PRE). No difference in the changes of the physiological profile variables (VO_2max_, sPeak, sVT2, sVT1 and RE) between sexes was detected with independent samples *t*‐test (Cohen's *d* = −0.24–0.46), and thus, sexes were combined for the analysis.

Changes in the physiological profile were tested with paired samples *t*‐test. For VO_2max_, sPeak, and sVT2 the comparisons were made with results from Visit 1, and for sVT1, RE, HR, CMJ, and MVC, they were compared to the first timepoint.

A two‐way repeated measures ANOVA (trial × time) was used to compare time‐dependent changes in sVT1, RE, HR, DFA‐α1, and CMJ between trials. If sphericity assumption was violated, Greenhouse–Geisser correction was used. If main effect was identified, a post hoc pairwise comparison was performed using paired samples *t*‐tests. Paired‐samples *t*‐tests were also used to compare VO_2max_, sPeak, sVT2, and VT2 between trials. Cohen's d was used to calculate effect sizes (ES).

ICCs (two‐way random, single measures, and absolute agreement) were calculated for absolute values of the variables as well as for the percentual changes in them. ICCs were interpreted as poor (< 0.5), moderate (0.5–0.75), good (0.75–0.9), and excellent (> 0.9) (Koo and Li 2016). Ninety‐five percent confidence intervals (CIs) were calculated for all ICCs.

Bland–Altman plots were created to assess the reliability of Δ%sPeak, Δ%VO_2max_, Δ%RE, Δ%HR, Δ%sVT2, and sVT1. Mean bias was calculated as follows: Test 2 – Test 1. Limits of agreement were calculated as follows: mean difference ± 1.96 x SD_differences_.

Intraindividual variation between trials was assessed using typical error of measurement (TE calculated as TE = SD_differences_· √2^−1^). Minimal detectable differences (MDCs) were calculated for all variables: MDC = 1.96·TE·√2. Within‐subject changes for absolute values of sVT1, RE, and CMJ were evaluated using coefficient of variation (CV), calculated as ([SD/mean] × 100%). CVs were used to assess whether reliability changed across different time points and were analyzed with the Friedman test, due to nonnormal distribution. All statistical analyses were conducted using SPSS (SPSS Inc., Chicago, IL, USA) and Microsoft Excel 2016 (Microsoft Corporation, Redmond, WA, USA).

## 3 Results

### 3.1 Characteristics of the Physiological Resilience Tests and Deterioration of the Physiological Profile

Characteristics of the physiological resilience test are presented in Table 2. Body masses did not differ in the beginning of the tests (73.6 ± 11.9 kg vs. 73.9 ± 11.9 kg, *p* = 0.115, and ICC = 0.999), and the changes in body masses did not differ between trials (−1.8 ± 0.6% vs. ‐ 1.8 ± 0.6%, *p* = 0.421, and ICC = 0.857).

**TABLE 2:** Characteristics (mean ± SD) of the physiological resilience tests including the incremental tests and moderate intensity running bouts.

Speed (km/h)	Distance (km)	VO_2_ (mL/kg/min)	%VO_2max_	%VO_2_ at VT1	%sVT1
10.7 ± 1.6	24.6 ± 3.7	36.3 ± 4.6	65.7 ± 4.5	90.2 ± 4.1	89.0 ± 0.2

*Note:* Oxygen uptake (VO_2_) results are from the first test as no difference between the two tests was detected (*p* > 0.895).

**FIGURE 2:**
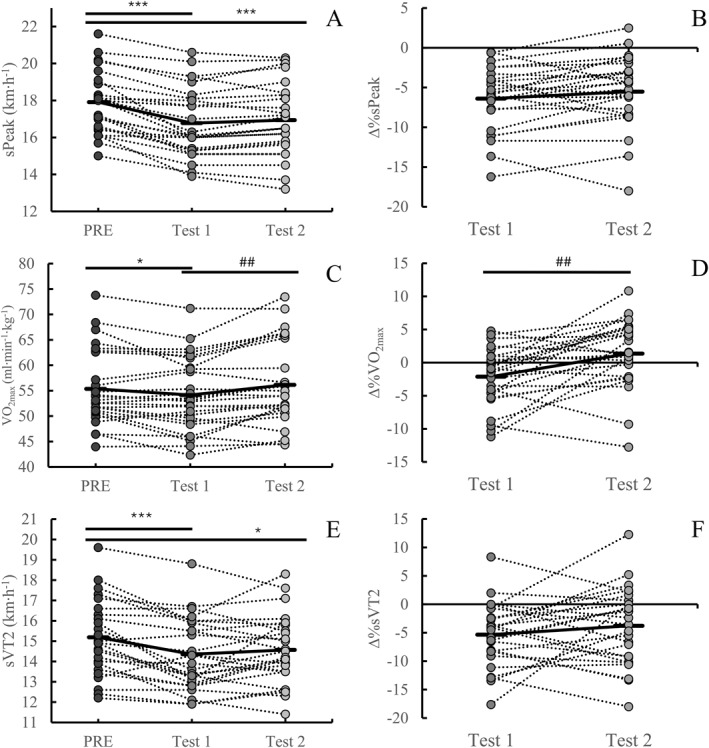
sPeak (2A), VO_2max_ (2C), and sVT2 (2E) measured in the VO_2max_ test during Visit 1 (PRE) and physiological resilience tests (Test 1 and Test 2), as well as changes in the physiological resilience tests compared to PRE (2B, 2D, and 2F). VO_2max_, maximal oxygen uptake; sPeak, maximal speed achieved in the VO_2max_ test; VT2, second ventilatory threshold. Differences are indicated by *p < 0.05 against PRE, **p < 0.01 against PRE, ***p < 0.001 against PRE, ^#^ *p* < 0.05 against Test 1, ^##^ *p* < 0.01 against Test 1, and ^###^ *p* < 0.001 against Test 1.

**FIGURE 3:**
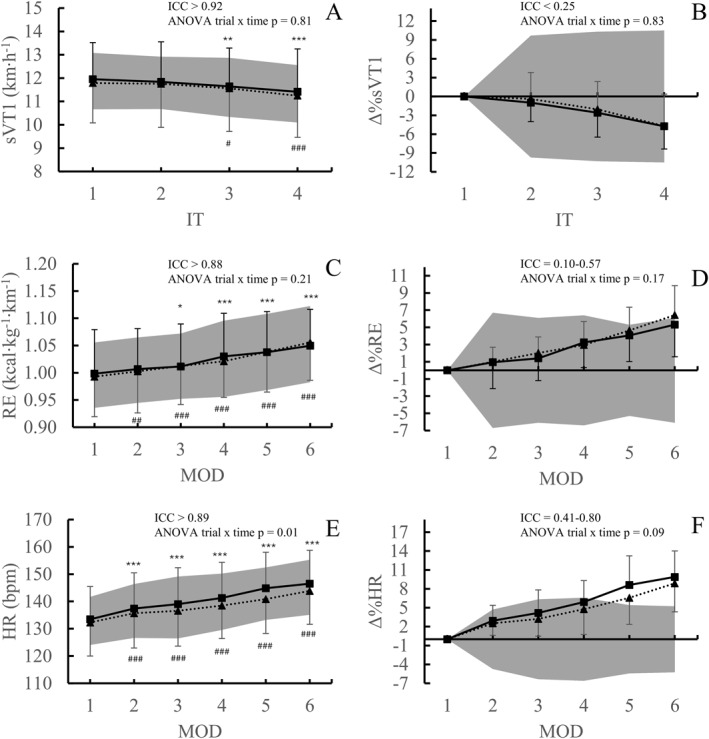
Mean (± SD) speed at first ventilatory threshold (sVT1), running economy (RE), and heart rate (HR) during different timepoints (3A, 3C, and 3E) and time course of the changes in the variables during the trial (3B, 3D, and 3F), during the first trial (solid line) and the second trial (dotted line). The gray area represents minimal detectable change (MDC). In Figures 3A, 3C, and 3E, it marks the MDC of different timepoints scaled to average of the timepoint. In Figures 3B, 3D, and 3F, MDC values at different time points are presented relative to the zero level to illustrate whether and when changes exceed the MDC. Differences are indicated as *p < 0.05 in first physiological resilience test versus the first timepoint, **p < 0.01 in first physiological resilience test versus the first timepoint, ***p < 0.001 in first physiological resilience test versus the first timepoint, ^#^ *p* < 0.05 in second physiological resilience test versus the first timepoint, *p* < 0.01 in second physiological resilience test versus the first timepoint, and ^###^< 0.001 in second physiological resilience test versus the first timepoint.

sPeak, sVT2, sVT1, and RE were all significantly deteriorated at the end of both tests compared with the nonfatigued state (*p* < 0.007) (Figures 2 and 3). VO_2max_ decreased only after the first test (*p* = 0.02) and not after the second (*p* = 0.16) (Figure 2). CMJ changed during the second test (*p* = 0.001) but not after the first (*p* = 0.05), whereas MVC decreased in the first test (*p* = 0.003) but not after the second (*p* = 0.06). HR increased from the start to the end in both tests (*p* < 0.001) (Figure 3), whereas DFA‐α1 showed no significant change in either test (*p* > 0.91).

### 3.2 Reliability of the Fatigued Physiological Profile

ICC analysis showed good to excellent reliability, for all endurance performance parameters (Table 3), as well as for HR (Figure 3), MVC, and CMJ (Table 4). DFA‐α1 showed moderate reliability at MOD1–3 (ICC = 0.58–0.69), poor reliability at MOD 4 (ICC = 0.37), and good reliability at MOD 6 (ICC = 0.77).

**TABLE 3:** The absolute values for measured endurance performance variables and associated reliability metrics.

Variable		Mean ± SD	ES	ICC	CV%	TE	TE%	MDC
VO_2max_ (l·min^−1^)	Test 1	3.9 ± 0.8	0.74	0.95 (0.79–0.98)	3.4	0.2	3.8	0.4
	Test 2	4.1 ± 0.8						
VO_2max_ (ml·kg^−1^ min^−1^)	Test 1	54.1 ± 7.3	0.67	0.90 (0.69–0.96)	3.3	2.1	3.7	5.7
	Test 2	56.1 ± 8.1						
sPeak (km·h^−1^)	Test 1	16.8 ± 1.8	0.35	0.97 (0.93–0.99)	1.7	0.3	1.9	0.9
	Test 2	16.9 ± 1.9						
VT2 (ml·g^−1^ min^−1^)	Test 1	48.8 ± 6.5	0.36	0.89 (0.76–0.95)	3.8	2.3	4.6	6.2
	Test 2	49.9 ± 7.0						
sVT2 (km·h^−1^)	Test 1	14.4 ± 1.7	0.22	0.83 (0.66–0.92)	4.0	0.7	4.8	1.8
	Test 2	14.6 ± 1.7						
VT1_IT1_ (ml·kg^−1^ min^−1^)	Test 1	39.4 ± 5.0	−0.48	0.85 (0.71–0.93)	4.1	2.0	5.2	5.6
	Test 2	38.9 ± 5.4						
VT1_IT2_ (ml·kg^−1^·min^−1^)	Test 1	40.0 ± 5.2	−0.10	0.93 (0.84–0.97)	3.2	1.7	4.2	4.6
	Test 2	39.9 ± 6.5						
VT1_IT3_ (ml·kg^−1^ min^−1^)	Test 1	40.1 ± 5.2	−0.51	0.89 (0.77–0.95)	4.0	2.0	5.0	5.6
	Test 2	39.6 ± 6.5						
VT1_IT4_ (ml·kg^−1^ min^−1^)	Test 1	40.0 ± 6.0	−0.33	0.92 (0.83–0.96)	3.4	1.8	4.5	4.9
	Test 2	39.7 ± 6.3						
sVT1_IT1_ (km·h^−1^)	Test 1	12.0 ± 1.6	−0.26	0.92 (0.84–0.97)	3.4	0.5	3.8	1.2
	Test 2	11.8 ± 1.7						
sVT1_IT2_ (km·h^−1^)	Test 1	11.8 ± 1.7	−0.14	0.95 (0.89–0.98)	2.9	0.4	3.5	1.1
	Test 2	11.8 ± 1.9						
sVT1_IT3_ (km·h^−1^)	Test 1	11.6 ± 1.6	−0.13	0.93 (0.85–0.97)	3.1	0.5	4.0	1.3
	Test 2	11.6 ± 1.8						
sVT1_IT4_ (km·h^−1^)	Test 1	11.4 ± 1.8	−0.26	0.94 (0.86–0.97)	3.3	0.5	4.0	1.2
	Test 2	11.2 ± 1.8						
RE_mod1_ (kcal·kg^−1^ km^−1^)	Test 1	1.00 ± 0.08	−0.18	0.93 (0.84–0.97)	1.7	0.02	2.2	0.06
	Test 2	0.99 ± 0.07						
RE_mod2_ (kcal·kg^−1^ km^−1^)	Test 1	1.01 ± 0.07	−0.13	0.92 (0.84–0.96)	1.7	0.02	2.1	0.06
	Test 2	1.00 ± 0.08						
RE_mod3_ (kcal·kg^−1^ km^−1^)	Test 1	1.01 ± 0.08	0.03	0.92 (0.84–0.96)	1.7	0.02	2.1	0.06
	Test 2	1.01 ± 0.07						
RE_mod4_ (kcal·kg^−1^ km^−1^)	Test 1	1.03 ± 0.08	−0.25	0.88 (0.76–0.95)	2.0	0.03	2.5	0.07
	Test 2	1.02 ± 0.07						
RE_mod5_ (kcal·kg^−1^ km^−1^)	Test 1	1.04 ± 0.07	0.02	0.89 (0.78–0.95)	1.7	0.03	2.4	0.07
	Test 2	1.04 ± 0.07						
RE_mod6_ (kcal·kg^−1^ km^−1^)	Test 1	1.05 ± 0.07	0.19	0.90 (0.78–0.95)	1.6	0.02	2.1	0.06
	Test 2	1.06 ± 0.07						

Abbreviations: CV, coefficient of variation; ES, effect size; ICC, intraclass correlation coefficient; MDC, minimal detectable change; RE, running economy; SD, standard deviation; sPeak, maximal speed achieved in the VO_2max_ test; TE, typical error of measurement; TE%, TE expressed as percentage of the average; Test 1, first physiological resilience test; Test 2, second physiological resilience test; VO_2max_, maximal oxygen uptake; VT1, first ventilatory threshold; VT2, second ventilatory threshold.

**TABLE 4:** The absolute values and changes in maximal voluntary contraction (MVC), countermovement jump (CMJ), and associated reliability metrics.

Variable		Mean ± SD	ES	ICC	CV%	TE	TE%	MDC
MVC PRE (N)	Test 1 Test 2	3419 ± 1138 3396 ± 1022	−0.09	0.97 (0.94–0.99)	4.5	185.7	5.4	514.7
MVC POST (N)	Test 1 Test2	3164 ± 973 3290 ± 961	0.53	0.96 (0.90–0.99)	4.7	171.5	5.3	475.3
CMJ_PRE_ (cm)	Test 1	32.3 ± 7.0	−0.24	0.97 (0.94–0.99)	3.7	1.1	3.5	3.2
	Test 2	32.0 ± 7.0						
CMJ_IT1_ (cm)	Test 1	33.0 ± 6.2	0.37	0.98 (0.95–0.99)	2.9	0.9	2.7	2.5
	Test 2	33.5 ± 6.0						
CMJ_IT2_ (cm)	Test 1	33.7 ± 6.4	−0.16	0.97 (0.94–0.99)	2.7	1.1	3.2	3.0
	Test 2	33.5 ± 6.5						
CMJ_IT3_ (cm)	Test 1	33.9 ± 6.7	−0.03	0.97 (0.95–0.99)	3.2	1.0	2.9	2.8
	Test 2	33.9 ± 6.5						
CMJ_IT4_ (cm)	Test 1	33.4 ± 6.9	0.27	0.94 (0.88–0.98)	6.2	1.6	4.6	4.3
	Test 2	34.0 ± 6.5						
Δ%MVC	Test 1 Test 2	−5.9 ± 10.3 −2.4 ± 7.1	0.41	0.50 (0.17–0.74)		6.2		17.1
Δ%CMJ_PRE‐1_	Test 1	2.7 ± 4.7	0.58	0.49 (0.12–0.74)		3.6		9.9
	Test 2	5.7 ± 5.9						
Δ%CMJ_PRE‐2_	Test 1	4.9 ± 5.5	0.08	0.50 (0.14–0.74)		3.9		10.8
	Test 2	5.4 ± 5.4						
Δ%CMJ_PRE‐3_	Test 1	5.3 ± 5.5	0.30	0.58 (0.26–0.78)		3.4		9.4
	Test 2	6.7 ± 5.0						
Δ%CMJ_PRE‐4_	Test 1	3.8 ± 8.1	0.40	0.49 (0.15–0.73)		6.3		17.5
	Test 2	7.4 ± 9.9						

Abbreviations: CV, coefficient of variation; CMJ, countermovement jump; ES, effect size; ICC, intraclass correlation coefficient; MDC, minimal detectable change; MVC, maximal voluntary contraction; SD, standard deviation; TE, typical error of measurement; TE%, TE expressed as percentage of the average; Test 1, first physiological resilience test; Test 2, second physiological resilience test.

All endurance performance variables showed low variability across timepoints (Table 3), with no effect of time on variability for sVT1 or RE detected (*p* > 0.68). CMJ, MVC (Table 4), and HR (CVs between 1.9% and 2.7% and TE% between 2.4% and 3.0%) had low variability as well. No effects of time were found for variability of CMJ (*p* = 0.06).

ANOVA analysis revealed no systematic differences between the physiological resilience tests for sVT1 (*p* = 0.23), RE (*p* = 0.71), CMJ (*p* = 0.65), and DFA‐α1 (*p* = 0.73), but differences were found for HR (*p* = 0.02). No trial × time interaction was detected for sVT1 (*p* = 0.81), RE (*P* = 0.21), or DFA‐α1 (*p* = 0.71), whereas interaction was found for HR (*p* = 0.01) and CMJ (*p* = 0.04). Paired comparisons revealed no statistically significant differences for sVT1, RE, and DFA‐α1 at any timepoint between the two tests (*p* > 0.05). For HR, significant differences were observed in every timepoint after MOD3 between the two tests (*p* < 0.03). No significant difference was found for sVT2 (*p* = 0.28) and sPeak (*p* = 0.08) between the two tests, but VO_2max_ differed between tests expressed in relative (*p* = 0.002) and absolute values (*p* < 0.001) (Figure 2). No statistically significant differences were observed for CMJ in any timepoint (*p* > 0.05). MVC differed in POST between trials (*p* = 0.01).

### 3.3 Reliability of the Physiological Resilience

ANOVA analysis showed no differences between the two trials at any timepoint for the Δ%sVT1 (*p* = 0.65)​ and Δ%RE (*p* = 0.32) (Figure 3), and no trial × time interaction was detected (*p* = 0.83 for Δ%sVT1 and *p* = 0.17 for Δ%RE). For Δ%HR, an effect of trial was found (*p* = 0.04), and the two tests differed at the fourth timepoint (*p* = 0.002), but there was no trial × time interaction (*p* = 0.09). ΔDFA‐α1 showed neither difference between the tests (*p* = 0.90) nor a trial × time interaction (*p* = 0.59). Δ%CMJ showed difference between the tests (*p* = 0.04), but no trial × time interaction was detected (*p* = 0.11). Paired comparisons revealed no differences between the two tests for Δ%sPeak (*p* = 0.09) or Δ%sVT2 (*p* = 0.23), but difference was found for Δ%VO_2max_ in relative (*p* = 0.002) and absolute values (*p* < 0.001) (Figure 2).

**FIGURE 4:**
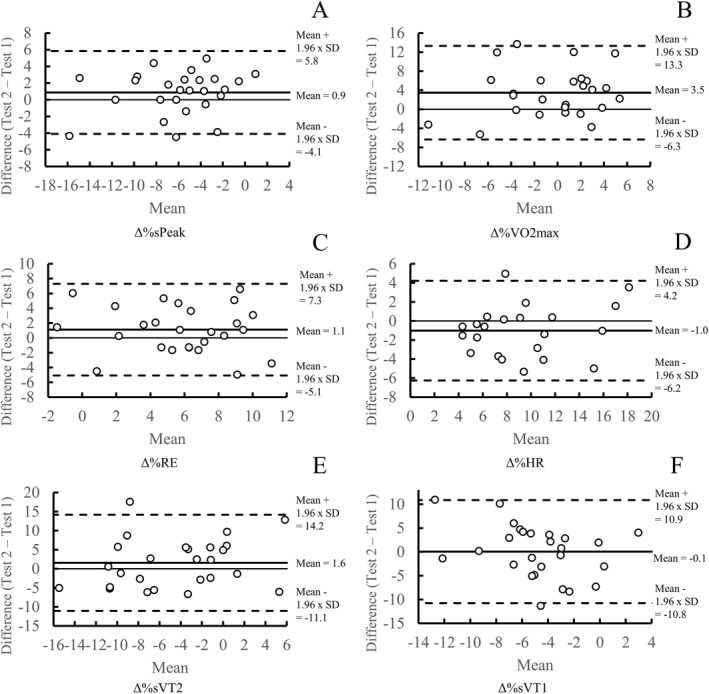
Bland–Altman plots for the changes (Δ%) in maximal speed of the incremental test (sPeak), maximal oxygen uptake (VO_2max_), running economy (RE), heart rate (HR), and speed at first and second ventilatory thresholds (sVT2 and sVT1). SD, standard deviation.

ICC analysis showed that Δ%sPeak had good reliability. Δ%VO_2max,_ Δ%sVT2, and Δ%sVT1 showed poor reliability in all timepoints (Table 5), and results for Δ%sVT1 were similar when sVT1 from the incremental treadmill test was used as the baseline (ICC = 0.06–0.10). Δ%RE showed poor reliability in the first half of the trial, but reliability improved in the latter stages, reaching moderate reliability in the final two MODs (Table 5). Δ%HR showed poor to moderate reliability in the first three timepoints (ICC = 0.41–0.58), but moderate to good reliability in the final two (ICC = 0.73–0.80). ΔDFA‐α1 showed poor reliability in the first four timepoints (ICC = 0.07–0.30), but moderate reliability in the last (ICC = 0.64). Δ%CMJ showed poor to moderate reliability overall and Δ%MVC showed moderate reliability (Table 4). TE for the changes in the variables were < 6.3 for all variables (Tables 4 and 5). Δ%HR showed low variability as well (TE 1.7–2.4). Mean bias showed that deterioration was lower during the second test versus the first test for all variables except RE (Figure 4).

**TABLE 5:** Percentage changes in the measured performance variables and associated reliability metrics.

Variable		Mean ± SD	ES	ICC	TE	MDC
Δ%VO_2max_ (absolute)	Test 1	−1.4 ± 4.5	0.76	0.38 (−0.01–0.67)	3.5	9.8
	Test 2	2.3 ± 5.3				
Δ%VO_2max_ (relative)	Test 1	−2.1 ± 4.3	0.70	0.36 (−0.01–0.65)	3.5	9.8
	Test 2	1.4 ± 5.0				
Δ%sPeak	Test 1	−6.4 ± 3.9	0.35	0.81 (0.61–0.91)	1.8	5.0
	Test 2	−5.5 ± 4.4				
Δ%sVT2	Test 1	−5.3 ± 5.6	0.24	0.46 (0.11–0.71)	4.6	12.6
	Test 2	−3.8 ± 6.8				
Δ%sVT1_IC1‐2_	Test 1	−1.0 ± 4.2	0.12	0.07 (−0.33–0.44)	3.6	10.0
	Test 2	−0.4 ± 3.0				
ΔsVT1_IC1‐3_	Test 1	−2.6 ± 4.4	0.10	0.23 (−0.17–0.57)	3.7	10.3
	Test 2	−2.0 ± 3.9				
Δ%sVT1_IC1‐4_	Test 1	−4.8 ± 5.1	0.01	0.25 (−0.16–0.58)	3.9	10.8
	Test 2	−4.7 ± 3.6				
Δ%RE_mod1‐2_	Test 1	0.9 ± 3.1	0.02	0.17 (−0.24–0.53)	2.3	6.4
	Test 2	1.0 ± 1.7				
Δ%RE_mod1‐3_	Test 1	1.4 ± 2.6	0.20	0.07 (−0.31–0.44)	2.2	6.1
	Test 2	2.0 ± 1.9				
Δ%RE_mod1‐4_	Test 1	3.3 ± 2.9	−0.09	0.36 (−0.03–0.66)	2.3	6.4
	Test 2	2.9 ± 2.7				
Δ%RE_mod1‐5_	Test 1	4.1 ± 3.0	0.22	0.57 (0.25–0.78)	1.9	5.3
	Test 2	4.6 ± 2.7				
Δ%RE_mod1‐6_	Test 1	5.3 ± 3.7	0.2	0.52 (0.18–0.75)	2.2	6.2
	Test 2	6.4 ± 3.4				

Abbreviations: CV, coefficient of variation; ES, effect size; ICC, intraclass correlation coefficient; MDC, minimal detectable change; RE, running economy; SD, standard deviation; sPeak, maximal speed achieved in the VO_2max_ test; TE, typical error of measurement; TE%, TE expressed as percentage of the average; Test 1, first physiological resilience test; Test 2, second physiological resilience test; VO_2max_, maximal oxygen uptake; VT1, first ventilatory threshold; VT2, second ventilatory threshold.

## 4 Discussion

The main findings of this study are as follows: (1) sPeak, sVT2, sVT1, and RE deteriorated during both trials, whereas VO_2max_ deteriorated only during first visit. (2) Measurements of VO_2max_, sPeak, sVT2, sVT1, and RE remained reliable in the fatigued state. (3) Deterioration of sPeak, RE, and HR were reliable, whereas deterioration of VO_2max,_ sVT2, and sVT1 were not reliable. Reliable measurement is essential when measuring time‐dependent changes after training interventions, and therefore, based on the results, deterioration of sPeak, RE, and HR should be used when measuring physiological resilience. Physiological profile can be measured with same reliability in a fatigued state as in a nonfatigued state.

### 4.1 Reliability of the Fatigued Physiological Profile

This is the first study to examine the test–retest reliability of VO_2max_, sPeak, RE, sVT2, and sVT1 during and after prolonged moderate‐intensity running. Our findings show similar reliability for VO_2max_ after prolonged moderate intensity running as what have been measured in a nonfatigued state (Bagger et al. 2003; Katch et al. 1982; Midgley et al. 2007). VO_2max_ was higher in the second trial, but the difference (average 2 mL·kg^−1^ min^−1^) between trials was similar to normal day‐to‐day variability of VO_2max_ (Knaier et al. 2019). Also, sPeak, which is highly related to VO_2max_, was similar between the two trials, which implies that higher VO_2max_ after the second trial is most likely related to the biological variability. Similarly, good reliability was obtained for sPeak, sVT2, sVT1, and RE in fatigued states. These are consistent with values reported in nonfatigued conditions (Blagrove et al. 2017; Lourenço et al. 2011; Shaw et al. 2013; Dickhuth et al. 2007; Saunders et al. 2004; Brisswalter and Legros 1994).

RE showed good to excellent reliability throughout the trial. Zanini et al. (2025b1) reported better reliability of EC during heavy intensity running than the current study. In the study by Zanini et al., the nutrition before the trial was standardized, which has been shown to result in better RE reliability in nonfatigued conditions (Blagrove et al. 2017; Shaw et al. 2013). In contrast, the present participants were only instructed to keep their nutrition and training consistent, but they were not strictly standardized. Reliability of HR and DFA‐α1 ranged from moderate to good. For HR, reliability was similar throughout the trial as previously measured in nonfatigued state (Blagrove et al. 2017; Saunders et al. 2004), but a difference appeared halfway through the trial, similarly to previous studies (Zanini et al. 2025b). This might be related to the participants being more accustomed to the test or possible differences in preparation for the test. The 3–4 bpm difference between trials falls within the normal day‐to‐day variability of HR (Achten and Jeukendrup 2003). Performing a third trial would give more insight whether this is biological variation or related to other factors. DFA‐α1 results were also in line with previous work (Van Hooren et al. 2023).

The measurement of the physiological profile appeared to remain reliable in fatigued state when compared to nonfatigued state, even though it significantly deteriorated. This is in line with previous studies in cycling (Clark et al. 2018; Mateo‐March et al. 2025) and RE in running (Zanini et al. 2025b). The fatigued state physiological profile has been proposed to be more important in endurance sports than the nonfatigued state, as competitions are decided after accumulated work (Noordhof et al. 2021). Also, the nonfatigued physiological profile does not, in all cases, separate athletes of different fitness levels as well as the fatigued state physiological profiles (Muriel et al. 2022). These findings demonstrate that physiological profile can be measured reliably after moderate intensity running and therefore can be used to monitor athletes.

### 4.2 Reliability of the Physiological Resilience

Physiological resilience is a relatively recent concept, of which research is increasing steadily. Many studies have measured the characteristic as a percentage change in selected variables (Nuuttila et al. 2024; Barrett and Maunder 2025; Gallo et al. 2024; Unhjem 2024; Zanini et al., 2025a, 2025b; Stevenson et al. 2022). The physiological profile deteriorated in the current study to a similar extent as previous studies have reported (Nuuttila et al. 2024; Barrett and Maunder 2025; Zanini et al., 2025a, 2025b) for all variables except VO_2max_ (Zanini et al., 2025a, 2025b). In the present analysis, Δ%sPeak was the most reliable metric when comparing ICC, TE, and MDC of the variables. sPeak is an important metric as it combines maximal aerobic capacity and running economy, and it has been shown to be highly associated with endurance running performance (Nuuttila et al. 2023). As Δ%sPeak is reliable and sPeak is associated with endurance performance, future research should investigate whether ability to sustain sPeak would be a valid metric to assess physiological resilience.

Regarding other outcomes, Δ%RE showed poor reliability in the first half of the trial, but its ICC improved in the last two timepoints and ended with good reliability. One possible reason for decline in RE during prolonged exercise is glycogen depletion in slow‐twitch muscle fibers and the subsequent recruitment of less economical fast‐twitch fibers, which worsen RE (Krustrup et al. 2004). It is possible that baseline muscle glycogen levels differed between trials in the current study, but the glycogen stores in Type I fibers may have been depleted to similar levels by mid‐run, leading to similar loss of RE by the end of the trials. Therefore, stricter nutritional standardization could enhance Δ%RE reliability earlier in the trial. Δ%HR has also been used to assess physiological resilience (Maunder et al. 2021; Matomäki et al. 2023; Smyth et al. 2022), and confirmation of its reliability is therefore important. Moreover, ΔDFA‐α1 has been shown to correlate with change in sLT1 (Nuuttila et al. 2024). As ΔDFA‐α1 appears to be a relatively reliable metric, it offers opportunities to develop future methods for assessing physiological resilience both inside and outside laboratory conditions.

In contrast to other metrics, Δ%VO_2max,_ Δ%sVT2, and Δ%sVT1 displayed poor reliability at every timepoint, with no improvement for sVT1 when the result from the VO_2max_ test was used as the baseline. A likely explanation for the poor reliability of Δ%sVTs and Δ%VO_2max_ is the larger variability in nonfatigued state (TE and MDC) than the deterioration in them and therefore a smaller signal‐to‐noise ratio. As the measurement is taken twice (PRE‐POST), the measurement error accumulates from both measurements and is thus greater for the changes. To achieve better reliability, the deterioration should exceed the noise of the measurement. By contrast, sPeak had a lower TE and MDC, as well as a larger decrement, implying better sensitivity to measure physiological resilience.

The poor reliability of the changes yields the question whether calculating percentage change is an appropriate method to assess and report physiological resilience, as done in previous studies. Previous studies with similar protocols (moderate‐intensity running at 90% of sVT1 or sLT1) have shown that sLT1 declined 5.5% after 90 min (Nuuttila et al. 2024) and 120 min reduced sVT1 by 6.2% (Barrett and Maunder 2025). These results have a similar magnitude, although slightly larger than the current study (4.7%–4.8%). Similarly, running in the heavy domain for 90 min reduced sLT1 by 3.6%, whereas extending the run to 120 min reduced the sLT1 by 6.6% (Zanini et al., 2025a, 2025b). Protocols with higher intensities have shown to decrease CP significantly more than lower intensity protocols despite higher total of work done at a lower intensity (Spragg et al. 2024). Different determination criteria for VT1 and VT2 might as well result in lower variability and better reliability in nonfatigued state (Pallarés et al. 2016; Dickhuth et al. 2007) and therefore improve the sensitivity to measure change in the VTs. These findings suggest that the reliable measurement of the Δ%VO_2max_, Δ%sVT1, and Δ%sVT2 might require either higher intensity, longer protocols, and/or measurement methods with a lower error to ensure that changes exceed the noise of the measurement. This is supported by the fact that the ICC for Δ%RE increased throughout the trial and was reliable once the change exceeded the TE and MDC. Similarly, ICC for Δ%sVT1 increased throughout the trial, although not sufficiently to demonstrate statistical significance. Future studies on physiological resilience measured during moderate‐intensity running may therefore consider using Δ%sPeak, Δ%RE, and HR drift to obtain reliable results, as these variables appear to be the most sensitive to change.

## 5 Limitations

The physiological resilience tests were conducted in the moderate‐intensity zone, which is not the typical zone for endurance running competitions and therefore may not reflect what occurs at racing speeds. For example, the study by Kyröläinen et al. (Kyröläinen et al. 2000) showed larger decreases in RE after a marathon (13.7% for EC), and Dressendorfer (Dressendorfer 1991) showed larger decrement in VO_2max_ (−6.2%) than the present study. To date, no studies have investigated the relationship among physiological resiliences across different intensity zones, so the generalizability of the current results remains unclear. As the intensity of the physiological resilience tests was VT1‐anchored, the determination of the VT1 might affect the results. Different methods to assess the first metabolic threshold might yield different results (Pallarés et al. 2016; Jamnick et al. 2018) and therefore affect the relative intensity of the exercise, which also has implications to the sex differences in physiological resilience (Meixner et al. 2026). Although subjects were instructed to keep their dietary and training habits the same before both physiological resilience tests, they were not standardized. This may have influenced the results as standardized diets have resulted in better reliability for RE (Blagrove et al. 2017; Shaw et al. 2013). The mouth rinse protocol before the VO_2max_ test in the physiological resilience tests might have potential day‐to‐day variability in the response and therefore might have affected the results of sPeak and VO_2max_. Although the methods determining VT1 and VT2 were reliable, alternative threshold assessment methods might yield lower error and thereby improve the reliability of Δ%sVT1 and Δ%sVT2. Future studies should investigate whether different methods to assess VTs, or other metabolic thresholds (e.g., LTs or critical speed), improve the reliability to measure the change in them. The menstrual cycle phase and hormonal contraceptive use were not controlled. As their effects on physiological resilience are unknown, they may have influenced the results. Additionally, due to the limited number of female participants, sex‐related effects to the reliability of physiological resilience cannot be excluded. Including a third physiological resilience test would have further strengthened the reliability assessment. Repeating the threshold and VO_2max_ tests would have provided better information on the typical variability of the measured variables in nonfatigued tests.

### 5.1 Future Perspectives and Practical Applications

In future, researchers should investigate whether longer and/or higher intensity protocols improve reliability of the measurement of physiological resilience. In addition, there is a need to evaluate whether standardizing nutrition prior and during trials affects reliability. Mechanistic studies are needed to improve our understanding of determinants and practical significance of physiological resilience. Based on the results of the current study, researchers may use sPeak, RE, and HR as measures of physiological resilience, when investigating properties of physiological resilience, as they appear to be the most robust. As resilience of VO_2max_ and VTs were less reliable, their use should be treated with greater caution in future studies, unless methodological refinement improves their sensitivity. One of the most important research questions is to find the most valid methods to assess physiological resilience related to competitions of different durations.

## 6 Conclusions

This is the first study to measure the test–retest reliability of the fatigued physiological profile, as well as the changes in the physiological profile during prolonged running (physiological resilience). The results showed good to excellent reliability for measurement of the physiological profile in a fatigued state. Although fatigued state physiological profile is important, it does not consider the fourth dimension of endurance performance, which is the deterioration of the profile. The reliability of physiological resilience was consistently good only in sPeak, whereas reliability was poor for Δ%VO_2max_, Δ%sVT2, and Δ%sVT1 with the current protocol. The reliability of HR drift and Δ%RE was moderate but increased throughout the trial. High variability of the measurement for VO_2max_ and VTs can cause poor reliability when measuring how they change during prolonged exercise, as the deterioration (signal) was weaker than the noise of the measurement. The results imply that the reliability of the measurement of a physiological profile is similar in a fatigued state to that of nonfatigued states. On the other hand, the findings suggest that the most reliable methods to assess physiological resilience during moderate‐intensity running are to utilize Δ%sPeak, Δ%RE, and Δ%HR.

## Funding

The study received a grant from Finnish Sports Research Foundation (Suomen urheilututkimussäätiö).

## Ethics Statement

The study was approved by the ethics committee of University of Jyväskylä (1581/13.00.04.00/2024).

## Conflicts of Interest

The authors declare no conflicts of interest.

## Supporting information


Supporting Information S1

## Data Availability

The data that support the findings of this study are available from the corresponding author upon reasonable request.
