# Supplementary material for: Test‐Retest Reliability of Physiological Resilience During and After Prolonged Moderate‐Intensity Running in Well‐Trained Runners
Source: Eur J Sport Sci. 2026 Apr 28;26(5):e70178. doi: 10.1002/ejsc.70178 (PMC13124681; doi:10.1002/ejsc.70178)
Supplement: Supplementary file 1 — Supporting Information S1 [file EJSC-26-e70178-s001.docx]

TABLE S1. The absolute values for oxygen cost and associated reliability metrics.

| Variable |  | Mean ± SD | ES | ICC | CV% | TE | TE% | MDC |
| --- | --- | --- | --- | --- | --- | --- | --- | --- |
| OC_mod1_ (ml∙kg^-1^∙km^-1^) | Test 1 | 198.4 ± 16.7 | -0.12 | 0.91 (0.81-0.96) | 1.8 | 4.8 | 2.4 | 13.4 |
|  | Test 2 | 197.6 ± 15.3 |  |  |  |  |  |  |
| OC_mod2_ (ml∙kg^-1^∙km^-1^) | Test 1 | 200.4 ± 15.7 | -0.07 | 0.90 (0.79-0.95) | 2.0 | 5.1 | 2.5 | 14.1 |
|  | Test 2 | 199.9 ± 16.2 |  |  |  |  |  |  |
| OC_mod3_ (ml∙kg^-1^∙km^-1^) | Test 1 | 202.5 ± 16.3 | 0.09 | 0.88 (0.75-0.95) | 2.0 | 5.4 | 2.7 | 15.1 |
|  | Test 2 | 203.2 ± 14.7 |  |  |  |  |  |  |
| OC_mod4_ (ml∙kg^-1^∙km^-1^) | Test 1 | 206.4 ± 16.7 | -0.15 | 0.84 (0.68-0.93) | 2.3 | 6.1 | 3.0 | 17.0 |
|  | Test 2 | 205.1 ± 14.1 |  |  |  |  |  |  |
| OC_mod5_ (ml∙kg^-1^∙km^-1^) | Test 1 | 209.4 ± 15.4 | 0.06 | 0.82 (0.63-0.91) | 2.1 | 6.7 | 3.2 | 18.5 |
|  | Test 2 | 210.0 ± 15.5 |  |  |  |  |  |  |
| OC_mod6_ (ml∙kg^-1^∙km^-1^) | Test 1 | 212.1 ± 13.6 | 0.21 | 0.82 (0.65-0.92) | 1.9 | 6.0 | 2.8 | 16.5 |
|  | Test 2 | 213.9 ± 14.9 |  |  |  |  |  |  |
|  |  |  |  |  |  |  |  |  |

Test 1: First physiological resilience test; Test 2: Second physiological resilience test; SD: standard deviation; ES: effect size; ICC: intraclass correlation coefficient; CV: coefficient of variation; TE: typical error of measurement; TE%: TE expressed as percentage of the average; MDC: minimal detectable change; OC: oxygen cost.

TABLE S2. Percentage changes in OC and associated reliability metrics.

| Variable |  | Mean ± SD | ES | ICC | TE | MDC |
| --- | --- | --- | --- | --- | --- | --- |
| Δ%OC_mod1-2_ | Test 1 | 1.1 ± 3.2 | 0.02 | 0.25 (-0.16-0.58) | 2.3 | 6.3 |
|  | Test 2 | 1.2 ± 1.9 |  |  |  |  |
| Δ%OC_mod1-3_ | Test 1 | 2.1 ± 2.7 | 0.25 | 0.18 (-0.20-0.52) | 2.3 | 6.3 |
|  | Test 2 | 2.9 ± 2.3 |  |  |  |  |
| Δ%OC_mod1-4_ | Test 1 | 4.1 ± 3.2 | -0.06 | 0.43 (0.06-0.70) | 2.4 | 6.7 |
|  | Test 2 | 3.9 ± 3.1 |  |  |  |  |
| Δ%OC_mod1-5_ | Test 1 | 5.6 ± 3.4 | 0.23 | 0.54 (0.21-0.76) | 2.2 | 6.0 |
|  | Test 2 | 6.3 ± 3.1 |  |  |  |  |
| Δ%OC_mod1-6_ | Test 1 | 7.1 ± 4.0 | 0.40 | 0.66 (0.37-0.83) | 2.3 | 6.3 |
|  | Test 2 | 8.4 ± 4.0 |  |  |  |  |

Test 1: First physiological resilience test; Test 2: Second physiological resilience test: SD: standard deviation; ES: effect size; ICC: intraclass correlation coefficient; CV: coefficient of variation; TE: typical error of measurement; TE%: TE expressed as percentage of the average; MDC: minimal detectable change; OC: oxygen cost.


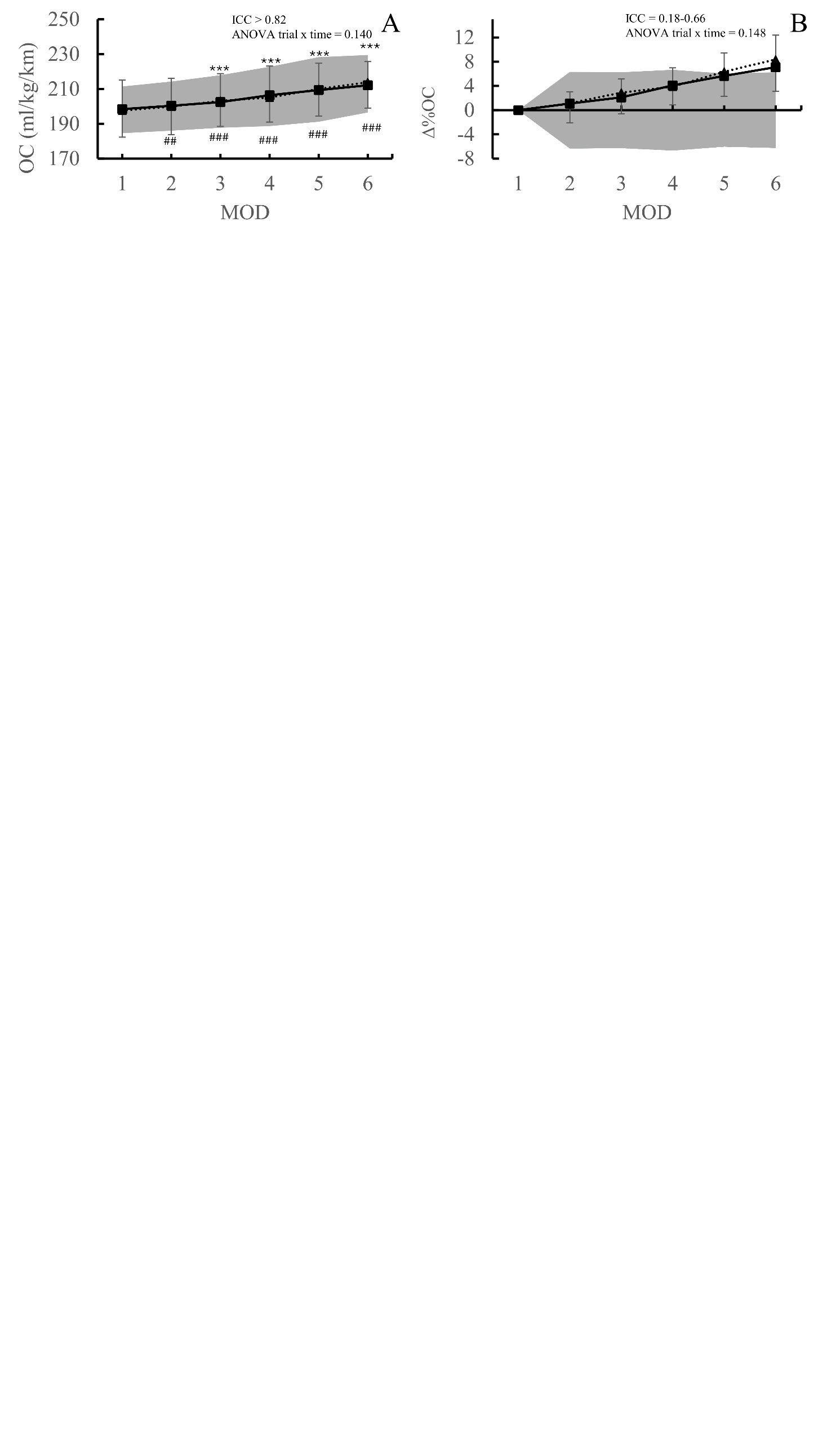


FIGURE S1. Mean (±SD) oxygen cost (OC) (panel A) and time course of the changes in OC during the trial (panel B), during the first trial (solid line), and the second trial (dotted line). The grey area represents minimal detectable change (MDC). In panel A it marks the MDC of different timepoints scaled to average of the timepoint. In panel B MDC of different timepoints is displayed relative to the zero level to illustrate if/when the change exceeds MDC. Differences are indicated as: * p < 0.05 in first physiological resilience test versus first timepoint, ** p < 0.01 in first physiological resilience test versus first timepoint, *** p < 0.001 in first physiological resilience test versus first timepoint, # p < 0.05 in second physiological resilience test versus first timepoint, p < 0.01 in second physiological resilience test versus first timepoint, and ### < 0.001 in second physiological resilience test versus first timepoint.
